# Alterations of monocyte NF-κB p65/RelA signaling in a cohort of older medical patients, age-matched controls, and healthy young adults

**DOI:** 10.1186/s12979-020-00197-7

**Published:** 2020-09-04

**Authors:** Juliette Tavenier, Line Jee Hartmann Rasmussen, Morten Baltzer Houlind, Aino Leegaard Andersen, Inge Panum, Ove Andersen, Janne Petersen, Anne Langkilde, Jan O. Nehlin

**Affiliations:** 1 Department of Clinical Research, Copenhagen University Hospital Hvidovre, 2650 Hvidovre, Denmark; 2 Department of Psychology and Neuroscience, Duke University, Durham, NC 27708 USA; 3 The Capital Region Pharmacy, 2730 Herlev, Denmark; 4 Department of Drug Design and Pharmacology, University of Copenhagen, 2100 Copenhagen, Denmark; 5 Department of Clinical Microbiology, Copenhagen University Hospital Hvidovre, 2650 Hvidovre, Denmark; 6 Emergency Department, Copenhagen University Hospital Amager and Hvidovre, 2650 Hvidovre, Denmark; 7 Department of Clinical Medicine, University of Copenhagen, 2200 Copenhagen, Denmark; 8 Center for Clinical Research and Prevention, Copenhagen University Hospital, 2000 Frederiksberg, Denmark; 9 Section of Biostatistics, Department of Public Health, University of Copenhagen, 1014 Copenhagen, Denmark

**Keywords:** Aging, Monocyte, NF-κB, Immunosenescence, Chronic inflammation, Inflammaging

## Abstract

**Background:**

Altered monocyte NF-κB signaling is a possible cause of inflammaging and driver of aging, however, evidence from human aging studies is sparse. We assessed monocyte NF-κB signaling across different aging trajectories by comparing healthy older adults to older adults with a recent emergency department (ED) admission and to young adults.

**Methods:**

We used data from: 52 older (≥65 years) Patients collected upon ED admission and at follow-up 30-days after discharge; 52 age- and sex-matched Older Controls without recent hospitalization; and 60 healthy Young Controls (20–35 years). Using flow cytometry, we assessed basal NF-κB phosphorylation (pNF-κB p65/RelA; Ser529) and induction of pNF-κB following stimulation with LPS or TNF-α in monocytes. We assessed frailty (FI-OutRef), physical and cognitive function, and plasma levels of IL-6, IL-18, TNF-α, and soluble urokinase plasminogen activator receptor.

**Results:**

Patients at follow-up were frailer, had higher levels of inflammatory markers and decreased physical and cognitive function than Older Controls. Patients at follow-up had higher basal pNF-κB levels than Older Controls (median fluorescence intensity (MFI): 125, IQR: 105–153 vs. MFI: 80, IQR: 71–90, *p* < 0.0001), and reduced pNF-κB induction in response to LPS (mean pNF-κB MFI fold change calculated as the log10 ratio of LPS-stimulation to the PBS-control: 0.10, 95% CI: 0.08 to 0.12 vs. 0.13, 95% CI: 0.10 to 0.15, *p* = 0.05) and TNF-α stimulation (0.02, 95% CI: − 0.00 to 0.05 vs. 0.10, 95% CI: 0.08 to 0.12, *p* < 0.0001). Older Controls had higher levels of inflammatory markers than Young Controls, but basal pNF-κB MFI did not differ between Older and Young Controls (MFI: 81, IQR: 70–86; *p* = 0.72). Older Controls had reduced pNF-κB induction in response to LPS and TNF-α compared to Young Controls (LPS: 0.40, 95% CI: 0.35 to 0.44, *p* < 0.0001; and TNF-α: 0.33, 95% CI: 0.27 to 0.40, *p* < 0.0001). In Older Controls, basal pNF-κB MFI was associated with FI-OutRef (*p* = 0.02).

**Conclusions:**

Increased basal pNF-κB activity in monocytes could be involved in the processes of frailty and accelerated aging. Furthermore, we show that monocyte NF-κB activation upon stimulation was impaired in frail older adults, which could result in reduced immune responses and vaccine effectiveness.

## Background

Twenty years ago, Franceschi et al. coined the term *inflammaging* in reference to the systemic, low-level increase in levels of inflammatory markers with age [1]. Since then, many studies have shown associations between this state of chronic inflammation and the development of frailty and most age-related chronic diseases including cancer, diabetes, cardiovascular and neurodegenerative disease [2–5]. Chronic inflammation has also emerged as a prognostic marker for mortality [6, 7]. However, the causes and effects of inflammaging are still a matter of debate and intense research [5]. In parallel with chronic inflammation, aging is associated with immunosenescence, i.e. the age-related decline in immune function [8]. Impaired innate immunity and monocyte function has been implicated in the reduced ability to fight viral and bacterial infections [9, 10] and could also have consequences for responses to vaccination in older adults [11]. Immunosenescence is thought to result in increased susceptibility to infections, a common cause of acute hospitalization and mortality in older adults [12–14].

The transcription factor nuclear factor kappa-light-chain-enhancer of activated B cells (NF-κB) is a master regulator of inflammatory responses in innate immune cells [15, 16]. Upon activation, NF-κB induces transcription of pro-inflammatory genes and activates inflammasomes such as NOD-, LRR- and pyrin domain-containing protein 3 (NLRP3). NF-κB (p65/RelA) also acts as a master regulator of the senescence-associated secretory phenotype (SASP) controlling both cell-autonomous and non-cell-autonomous aspects of the senescence program [17]. In inflammaging, the elevated circulating levels of interleukin (IL)-6, IL-8, and tumor necrosis factor-α (TNF-α) have been attributed to increased NF-κB activity in innate immune cells such as monocytes [18]. Much of this evidence is based studies from animal models, and there is little direct evidence of a link between monocyte NF-κB signaling and chronic inflammation in human aging studies. Furthermore, due to its central role in the regulation of immune responses, altered NF-κB signaling could also be involved in the reduced monocyte responsiveness observed with age.

**Fig. 1:**
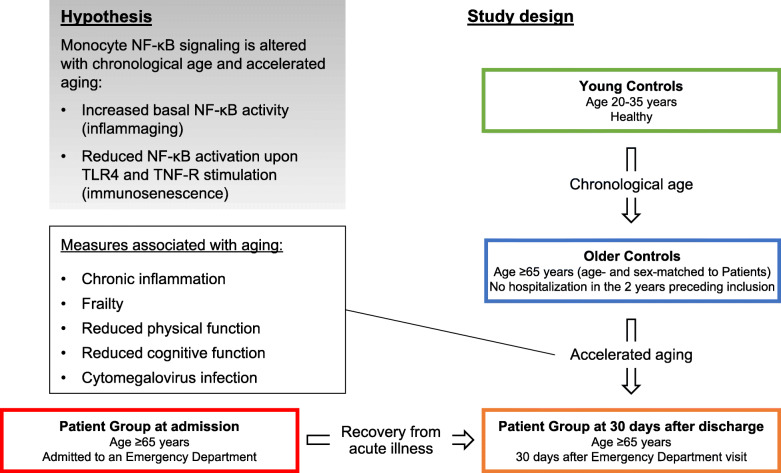
Study design and hypothesis. We evaluated the effect of chronological age on monocyte NF-κB signaling (p65/RelA S529 basal phosphorylation levels and induction of phosphorylation upon TLR4 and TNF-R stimulation) by comparing Young to Older Controls. We evaluated the role of monocyte NF-κB dysregulation in the aging process by comparison of two populations of older individuals with different aging trajectories: recently hospitalized Patients (hypothesized to exhibit signs of accelerated aging) and age-matched Older Controls. Furthermore, we explored whether the possible role of altered monocyte NF-κB signaling in aging was mediated by chronic inflammation, frailty, physical and cognitive decline. We also tested whether CMV-infection was a confounder for the association of monocyte NF-κB signaling with aging. Abbreviations: CMV: cytomegalovirus; TLR: Toll-like receptor; TNF-R: tumor necrosis factor receptor.

Much of the work examining changes to monocyte function and signaling in aging has compared healthy older adults with healthy younger adults [19–23]. However, aging is a highly heterogeneous process and there is a considerable variability in the rate of aging across older adults [24]. Studying older individuals on different trajectories of aging could be beneficial to gain a better understanding of dysregulation of inflammatory and immune responses in older adults and their potential role in the aging process. This knowledge could also lead to the development of improved anti-viral and anti-bacterial treatments and to generating lifesaving vaccines that are also effective in older populations. It has been suggested that age-related chronic diseases and geriatric syndromes such as frailty could be considered expressions of accelerated aging [25]. Frailty is characterized by the loss of physiological reserve capacity as well as an increased vulnerability to stressors, and is therefore highly prevalent among acutely hospitalized older adults [26]. Thus, we hypothesized that older adults requiring an Emergency Department visit could represent a frailer population on a trajectory of accelerated aging compared to an age-matched population without recent hospitalization (Fig. 1).

Here, we aimed to evaluate the effects of chronological age on monocyte NF-κB signaling (both basal levels and activation upon stimulation with LPS or TNF-α) by comparing healthy older adults to healthy young adults. Furthermore, we aimed to investigate whether dysregulated NF-κB signaling in monocytes is involved in the aging process by comparing two groups of older adults with different aging trajectories. Thus, we designed this study to include three groups: one group of healthy young adults and two groups of older adults. The two groups of older adults consisted of (i) patients with a recent unplanned Emergency Department (ED) visit, and (ii) age- and sex-matched older adults without any recent hospitalization (Fig. 1). We contemplated that, once they have recovered from the acute illness, it would become apparent that the older patients exhibited evident signs of accelerated aging such as frailty, chronic inflammation, decreased physical and cognitive function compared to age- and sex-matched older adults without recent hospitalization. Finally, we aimed to explore how dysregulated monocyte NF-κB signaling contributes to accelerated aging by investigating its associations with inflammation, frailty, and physical and cognitive decline in older adults.

## Methods

### Study description

This study uses data collected as part of *FAM-CPH*, an observational cohort study aiming to investigate mechanisms of biological aging and chronic inflammation, as well as malnutrition, and medication in acutely ill and healthy older adults as well as healthy young adults. Previous results from *FAM-CPH* regarding drug prescription have been published elsewhere [27]. *FAM-CPH* was approved by the Health Research Ethics Committee for the Capital Region of Denmark (H-16038786), the Danish Data Protection Agency (AHH-2016-067) and was registered at Clinicaltrials.gov (NCT03052192). The study was conducted in accordance with the Declaration of Helsinki, and all participants gave written informed consent.

#### Study participants

Three groups of participants were recruited between November 2016 and September 2019: (i) Patient Group: older adults admitted to the ED due to acute illness; (ii) Older Controls: older adults matched on age and sex with participants from the Patient Group, but with no hospital admissions in the 2 years preceding inclusion; (iii) Young Controls: healthy adults aged 20–35 years.

##### Patient group

Inclusion criteria were: ≥65 years of age, acutely admitted to the ED, able to speak and understand Danish, caucasian. Exclusion criteria were: inability to cooperate cognitively, terminal illness or illness requiring isolation. For this *FAM-CPH* substudy, we further excluded patients with autoimmune disease or current cancer diagnosis, or patients who were prescribed systemic immunosuppressive or anti-inflammatory medication. Patients were included during their admission to the ED at Copenhagen University Hospital Hvidovre, Denmark. Blood samples and data were collected upon hospital admission, as well as in the patient’s home 30 days after discharge and 1 year after the first follow-up visit.

##### Older controls

Participants in the Older Control group were included based on 1:1 matching on age and sex with participants form the Patient Group at the 30-day follow-up. We initially aimed to match Older Controls and Patients by municipality of residence. Due to a lower than expected response rate of Older Controls, this could not be achieved. However, all Patients and Older Controls resided in the same group of municipalities corresponding to the catchment area of Copenhagen University Hospital Hvidovre. Inclusion criteria were: ≥65 years of age, caucasian, no hospital admissions in the 2 years preceding inclusion. Exclusion criteria were: acute hospital admissions in the 2 years preceding inclusion, current treatment with immunosuppressive or biological medication, and cancer or auto-immune illness (see the full list of diagnoses and medication in Additional File 1; Table S1 and S3). Potential participants were identified by The Danish Health Data Authority via the Danish Civil Registration System and the Danish National Patient Registry and were invited to participate by letter. Older Controls were asked to visit the Clinical Research Centre at Copenhagen University Hospital Hvidovre, for blood and data collection at inclusion and at a follow-up visit after 1 year.

##### Young controls

Inclusion criteria were: age between 20 and 35 years, caucasian, no hospital admission due to chronic or severe illness in the 5 years preceding inclusion (except childbirth, abortion, appendicitis, poisoning, trauma, or concussion). Exclusion criteria were: auto-immune or chronic illness (see the full list of diagnoses and medication in Additional File 1; Table S2 and S3). Young Controls were recruited by advertisement and asked to visit the Clinical Research Centre for blood and data collection at inclusion and at a follow-up visit after 30 days.

The present study includes data from the Patient Group at hospital admission and at the follow-up visit 30 days after discharge, and from the Older and Young Control groups at their respective inclusion visits. One-year mortality status for participants from the Patient Group and Older Control group is also reported.

### Whole blood stimulations and flow cytometry

#### Whole blood stimulations

Heparinized whole blood was rested for 15 min at 37 °C prior to stimulation and subsequent freezing. Samples were then incubated for 15 min at 37 °C with PBS (control), 1 μg/mL lipopolysaccharides (LPS) 055:B5 (Sigma-Aldrich, St. Louis, MO, United States), or 100 ng/mL recombinant human TNF-α (BioLegend, San Diego, CA, USA), or left unstimulated. Stimulated and unstimulated samples were divided into 1 mL aliquots and incubated with 1.4 mL Proteomic Stabilizer (Smart Tube Inc., Palo Alto, CA, USA) at room temperature for fixation, followed by storage at − 80 °C until thawing and antibody staining.

#### Antibody staining

Whole blood aliquots stored at − 80 °C were thawed in a 10 °C water bath, and red blood cells were lysed using Thaw-Lyse Buffer (Smart Tube). Peripheral blood leukocytes were then washed twice in cell staining media (2 mM EDTA (Life Technologies, Carlsbad, CA, United States) and 0.5% bovine serum albumin (Miltenyi Biotec, Bergisch Gladbach, Germany) in PBS). Cells were permeabilized in 2 mL cold 100% methanol for 10 min at 4 °C and stored at − 80 °C overnight. Cells were then washed twice in cell staining media and resuspended in 10% heat-inactivated human serum in PBS for 15 min at room temperature to block non-specific Fc receptor binding. Cells were stained for 45 min at room temperature with the following titrated antibody cocktail: anti-CD66b (G10F5) AlexaFluor700 (BioLegend), anti-HLA-DR (L243) Brilliant Violet 711 (BioLegend), anti-CD14 (61D3) eFluor450 (eBioscience, San Diego, CA, USA), anti-CD16 (CB16) FITC (eBioscience), anti-NF-κB p65 (pSer529) (K10–895.12.50) PE-CF594 (BD Biosciences), anti-NLRP3 (REA668) APC (Miltenyi Biotec). Cells were then washed twice with cell staining media and analyzed on an LSRFortessa (BD Biosciences) flow cytometer. Matching isotype (AlexaFluor700 IgM (RnD Systems, Minneapolis, MN, USA), Brilliant Violet 711 IgG2a κ (BioLegend), eFluor450 IgG1 κ (eBioscience), FITC IgG1 κ (eBioscience), PE-CF594 IgG2b κ (BD Biosciences), and APC IgG2a κ (Miltenyi Biotec)) and unstained controls were included for each sample.

#### Flow cytometry

For each sample, 50,000 CD14 CD16 monocyte events were recorded on an LSRFortessa flow cytometer (BD Biosciences). Samples from matched Patient-Older Control pairs were assessed in the same batch. Compensations were generated for each fluorochrome using CompBeads (Anti-Mouse Ig,κ; BD Biosciences) and anti-mouse conjugated antibodies. Data were analyzed using FlowJo v10 (BD Biosciences).

In addition, total monocyte numbers from fresh whole blood samples were routinely measured by the Department of Clinical Biochemistry, Copenhagen University Hospital Hvidovre on a Sysmex XN-9000 analyzer (Sysmex Europe GmbH, Norderstedt, Germany).

#### Flow cytometry data analysis

The gating strategy for monocytes and monocyte subsets was as follows: monocytes from whole blood were identified using their high forward/side scatter properties. Single cells were selected using a side scatter height versus area plot. Next, granulocytes were excluded based on positive CD66b staining, and HLA-DR positive monocytes were selected. Monocytes were then subtyped into three subsets based on CD14 and CD16 expression: classical (CD14^++^CD16^−^), intermediate (CD14^++^CD16^+^), and non-classical (CD14^+^CD16^+^) monocytes [28]. NF-κB signaling was assessed as (i) basal NF-κB p65 phosphorylation (pNF-κB, p65/RelA S529), reported as the median fluorescence intensity (MFI); and (ii) the induction of NF-κB phosphorylation in response to stimulation with LPS or TNF-α, reported as fold change calculated as the transformed ratio of pNF-κB MFI from the stimulated samples and control sample on the log10 scale: log10(MFI of LPS or TNF-α) – log10(MFI of PBS control). We also report expression of HLA-DR and NLRP3 inflammasome.

Although 52 Patients were included, heparinized whole blood samples were only available for 48 Patients at admission. Due to logistical and technical reasons, we were only able to analyze unstimulated (basal) samples for subset distribution for: 42/48 Patients at admission, and 50/52 at follow-up, 52/52 Older adults, and 60/60 Young Controls. LPS stimulation experiments were performed for: 36/48 Patients at admission, 50/52 at Patients at follow-up, 52/52 Older Controls, and 20/60 Young Controls. TNF-α stimulation experiments were performed for: 16/48 Patients at admission, 31/52 at Patients at follow-up, 52/52 Older Controls, and 20/60 Young Controls.

### Plasma biomarker measurements

Blood samples were collected by venipuncture in anti-coagulant EDTA containing tubes for plasma preparation.

Plasma levels of the chronic inflammation marker soluble urokinase plasminogen activator receptor (suPAR) for the Patient Group and Older Controls were measured by the Department of Clinical Biochemistry at Copenhagen University Hospital Hvidovre using the suPARnostic (ViroGates A/S, Birkerød, Denmark) enzyme-linked immunosorbent assay (ELISA). Plasma suPAR levels for Young Controls were measured by the Clinical Research Centre at Copenhagen University Hospital Hvidovre using the suPARnostic ELISA.

Levels of IL-1β, IL-6, IL-10, and TNF-α were measured using High Sensitivity Magnetic Luminex assays (RnD Systems, Minneapolis, MN, USA). IL-18 levels were measured using Magnetic Luminex assays (RnD Systems) on the Luminex 200 System (Luminex Corporation, Austin, TX, USA). Growth differentiation factor 15 (GDF15), macrophage inflammatory protein-1α (MIP-1α) and IL-8 levels were measured using Human Quantikine ELISA assays (RnD Systems). All assays were performed by the Clinical Research Centre, Copenhagen University Hospital Hvidovre according to the manufacturer’s instructions. Samples were analyzed in duplicates and if the intra-assay coefficient of variation was greater than 20% for Luminex and 10% for ELISA, the samples were re-analyzed. Samples from the Patient Group, Older and Young Controls were distributed between assays, but admission and follow-up samples from the same Patient and the sample for their matched Older Control were analyzed in the same assay. Values below the detectable range of the assays were assigned values corresponding to the lowest detectable level, this was the case for 92.7, 32.5, 88.2, 0.9, 0, and 0% of samples for IL-1β, IL-6, IL-10, TNF-α, IL-18, and GDF15 respectively. The average intra-assay coefficients of variation were 16.4, 8.1, 10.2, 4.4, 2.8, and 3.0% for IL-1β, IL-6, IL-10, TNF-α, IL-18, and GDF15 respectively.

### Background data and aging measures

Current smoking status was obtained via interview. Anthropometric data included height (meters), weight (kg), and body mass index (BMI; kg/m^2^).

Data on the reason for hospitalization reported by the patients (categorized as cardiovascular symptoms, infections, respiratory symptoms, fall, or other reason), length of hospital stay, and cause of death for the Patient Group were collected from the patients’ electronic health records.

We collected data on aging measures, including frailty, physical and cognitive function. Frailty was assessed using the frailty index (FI)-OutRef. FI-OutRef was developed in an acute care setting [29] and is calculated as the number of biomarkers outside of reference range for 17 blood biochemistry biomarkers (alanine aminotransferase, albumin, alkaline phosphatase, bilirubin, blood urea nitrogen, coagulation factors II, VII and X, C-reactive protein (CRP), creatinine, hemoglobin, lactate dehydrogenase, mean corpuscular hemoglobin concentration, mean corpuscular volume, neutrophils, potassium, sodium, thrombocytes, white blood cell count). The biomarkers were routinely measured by the Department of Clinical Biochemistry, Copenhagen University Hospital Hvidovre.

Physical function was assessed using hand grip strength, leg strength, and gait speed. Hand grip strength (kg) was measured in the dominant hand (or strongest hand for participants reporting loss of strength in the dominant hand due to surgery or arthritis) using a handheld dynamometer (model Digi-II, Saehan Corp., Masan, South Korea). The highest grip strength measurement out of a minimum of three and maximum of five attempts was used. Leg strength was assessed using the 30 s chair stand test and reported as the number of repetitions. Gait speed (m/s: usual gait speed) was assessed using the 4-m gait speed test. Participants were directed to walk at their normal pace, and the fastest of two attempts was used.

Cognitive function was assessed using the Mini Mental State Examination (MMSE) [30] and Trail Making Test A and B [31]. For the Patient Group, these tests were not administered at hospital admission.

### Cytomegalovirus status

Cytomegalovirus (CMV) IgG levels were measured by electrochemiluminescence on a COBAS platform at the Department of Microbiology, Copenhagen University Hospital Hvidovre. Samples with measurements below the detectable range of the assay (0.150 U/mL) were assigned values of 0.150 U/mL. Samples with values below 0.5 U/mL were considered negative for CMV infection (seronegative).

### Statistical analyses

For descriptive statistics, continuous data are presented as median and interquartile range (IQR) or mean and standard deviation (SD) when normally distributed. Categorical data are presented as number of participants and percentage. To assess differences between the Older and Young Controls, we used Student’s t-test, Wilcoxon rank-sum test, Chi-square or Fisher’s exact test where appropriate. To assess differences between Patients at admission and follow-up, and between Patients at follow-up and Older Controls, we used paired t-test, Wilcoxon signed-rank test, Chi-square or Fisher’s exact test where appropriate.

We assessed differences in monocyte phenotypes and NF-κB signaling between the Older and Young Controls using Student’s t-test or Wilcoxon rank-sum test where appropriate. We assessed differences in monocyte phenotypes and NF-κB signaling between Patients at admission and follow-up, and between Patients at follow-up and Older Controls, using paired t-test or Wilcoxon signed-rank test where appropriate. Mean and 95% confidence intervals (95% CI) are reported for t-tests, and median and IQR are reported for Wilcoxon tests.

We analyzed the associations of NF-κB signaling with age, plasma biomarkers, frailty, and physical and cognitive function in the Patient Group at follow-up and in the Older Controls using simple linear regressions. Plasma biomarker variables were log-transformed, and estimates and 95% CI were back-transformed. Estimates (β) and 95% CI indicate the change in the dependent variable—expressed in the original unit, or in percent change for plasma biomarkers—per 10% increase in pNF-κB MFI. Furthermore, we assessed the association of CMV infection with pNF-κB MFI in the Patient Group and Older Controls using simple linear regressions. pNF-κB MFI was log-transformed, and beta estimates (β) and 95% CI were back-transformed and are shown as percent difference in pNF-κB MFI for CMV IgG seropositive individuals compared to seronegative individuals.

We assessed the influence of monocyte NF-κB phosphorylation on the probability of belonging to the Patient Group (i.e. having a recent unplanned ED visit) using conditional logistic regressions. We computed the odds ratio (OR) estimates and 95% CI for belonging to the Patient Group per 10% increase of basal pNF-κB MFI in an unadjusted logistic regression model. Then, we adjusted the model individually for each of the plasma biomarkers, frailty, physical and cognitive function, and CMV-infection – all markers that we hypothesized were mediators or confounders for the observed effect.

Analyses were performed using Statistical Analysis Systems (SAS) Enterprise Guide version 7.1 (SAS Institute, Cary, NC, USA). Graphs were made using GraphPad Prism version 8 (GraphPad Software, San Diego, CA, USA). A *p*-value < 0.05 was considered to indicate a statistically significant difference.

## Results

### Baseline characteristics of the study participants

In total, 128 Patients, 52 Older Controls, and 60 Young Controls were included in *FAM-CPH*. Out of the 128 included Patients, one withdrew consent, 8 had died and 23 had dropped-out at the 30-day follow-up. Out of the 96 Patients remaining at follow-up, 42 were further excluded from this substudy due to autoimmune disease, cancer, prescriptions for systemic immunosuppressive or anti-inflammatory medication, or no blood samples available for flow cytometry analysis. Thus, 54 Patients could be age- and gender-matched with Older Controls. We invited Older adults matched on age, sex, and municipality to the 54 Patients to participate in the study. Due to a lower response rate than anticipated, we were not able to fulfill the criteria for matching on municipality, but we included 52 Older Controls matched 1:1 on age and sex to 52 of the Patients. One Young Control was excluded from analyses due to reporting current illness and had elevated CRP levels, leaving 59 Young Controls available for analyses. Thus, in total, data from 52 Patients, 52 age- and sex-matched Older Controls, and 59 Young Controls were analyzed. The median age for Patients and Older Controls was 75 years, and 48% of participants were women. Young Controls had a median age of 26 years and 49% were women (Table 1).

**Table 1:** Characteristics of the study participants

	Patient Group				Older Controls		Young Controls	
	Admission to the ED		30-day follow-up		Inclusion		Inclusion	
	n	median (IQR) or n (%)	n	median (IQR) or n (%)	n	median (IQR) or n (%)	n	median (IQR) or n (%)
**Demographics and lifestyle**								
Age (years)^1^	52	74.8 (70.6–81.8)	52	74.9 (70.7–82.0)	52	74.8 (70.7–81.9)	59	26.4 ^b^ (24.0–29.0)
Sex (women)^2^	52	25 (48.1)		–	52	25 (48.1)	59	29 (49.2)
Smoking^3^–Daily smoker	52	6 (11.5)		–	52	4 (7.7)	59	0 ^b^ (0)
Occasional smoker		0 (0)		–		0 (0)		4 (6.8)
Former smoker		34 (65.4)		–		29 (55.8)		13 (22.0)
Never smoked		12 (23.1)		–		19 (36.5)		42 (71.2)
BMI (kg/m^2^)^1^	52	26.8 (23.0–31.5)	52	26.4 (22.2–31.7)	51	25.9 (22.7–27.9)	59	22.8 ^b^ (21.7–24.2)
**Hospitalization**								
Length of stay (days)	52	1 (1–4)		–		–		–
Reason for hospitalization	52							
Cardiovascular symptoms		16 (30.1)		–		–		–
Infection		11 (21.2)		–		–		–
Respiratory symptoms		10 (19.2)		–		–		–
Fall		5 (9.6)		–		–		–
Other symptoms		10 (19.2)		–		–		–
**Plasma biomarkers**								
CRP (mg/L)^4^	52	6.2 (2.5–48.0)	52	3.2 ^ab^ (1.3–8.7)	51	1.2 (0.5–2.4)	59	0.4 ^b^ (0.3–0.9)
suPAR (ng/mL)^4^	50	3.2 (2.9–4.8)	52	3.3 ^b^ (2.6–4.7)	52	2.6 (2.2–3.1)	59	2.0 ^b^ (1.8–2.5)
IL-6 (pg/mL)^4^	49	2.4 (0.8–6.3)	52	0.8 ^ab^ (0.6–1.6)	52	0.6 (0.3–0.9)	59	0.3 ^b^ (0.3–0.3)
IL-18 (pg/mL)^4^	49	336.1 (213.0–434.2)	52	304.0 ^b^ (197.7–404.1)	52	216.9 (167.0–344.4)	59	183.8 ^b^ (144.4–239.0)
TNF-α (pg/mL)^4^	49	9.0 (7.0–13.3)	52	9.4 ^b^ (7.3–14.0)	52	8.2 (6.4–9.8)	59	5.3 ^b^ (4.4–6.1)
GDF15 (pg/mL)^4^	49	1873.5 (1177.1–2729.9)	52	1562.5 ^ab^ (1043.8–2186.2)	52	1003.9 (829.4–1297.0)	20	288.1 ^b^ (232.7–311.2)
**Frailty**								
FI-OutRef^1^	50	4.3 (1.3–6.4)	52	3.2 ^b^ (1.1–5.3)	50	1.0 (0.0–2.0)	59	0.0 (0.0–1.0)
**Physical function**								
4-m gait speed (m/s)^1^	37	0.75 (0.62–0.93)	50	0.79 ^ab^ (0.61–1.00)	52	1.25 (1.11–1.33)	59	1.43 ^b^ (1.33–1.54)
Hand grip strength (kg)^1^	51	22.5 (17.6–37.2)	51	25.4 ^ab^ (18.9–37.2)	52	30.8 (22.3–39.7)	59	39.8 ^b^ (33.8–50.4)
Chair stand test (repetitions)^1^	27	11 (9–14)	41	12 ^ab^ (9–14)	52	13 (11–16)	59	24 ^b^ (20–28)
**Cognitive function**								
MMSE (points)^1^		–	47	29 (26–30)	52	29 (27–30)		–
Trail making test A (seconds)^4^		–	47	50.0 ^b^ (37.0–69.0)	51	37.8 (29.0–54.6)	59	17.4 ^b^ (15.0–21.5)
Trail making test B (seconds)^4^		–	46	149.9 ^b^ (100.0–246.0)	49	86.9 (67.0–113.4)	59	44.5 ^b^ (38.7–53.5)
**Mortality**								
1-year mortality^3^			52	4 (7.7)	52	0 (0)		–
**Cytomegalovirus status**								
Seropositive^3^		–	51	31 (60.8)	52	37 (71.2)	59	40 (67.8)
Anti-CVM IgG titer^4^ (U/mL)		–	51	127.2 (0.2–461.5)	52	251.4 (0.2–424.2)	59	162.2 (0.2–410.1)

^1^ Paired t-test or Student’s unpaired t-test

^2^ Chi-square test

^3^ Fisher’s exact test or McNemar’s test

^4^ Wilcoxon signed-rank test or Wilcoxon rank-sum test

^a^ *p* < 0.05 for comparison with admission values

^b^ *p* < 0.05 for comparison with Older Controls

Abbreviations: *BMI* body mass index; *CRP* C-reactive protein; *ED* emergency department, *FI-OutRef* frailty index OutRef; *GDF15* growth differentiation factor 15; *IL* interleukin; *MMSE* mini mental state examination; *suPAR* soluble urokinase plasminogen activator receptor; *TNF-α* tumor necrosis factor-alpha

First, we assessed whether Patients had recovered from acute illness at the 30-day follow-up. Patients were only admitted for a median duration of 1 day (Table 1). At 30 days after discharge, levels of the acute inflammation markers IL-6 (*p* < 0.0001) and CRP (*p* = 0.0006) were significantly lower than at admission (Table 1). GDF15 levels had also decreased at follow-up (*p* = 0.0005). Furthermore, physical function also improved at follow-up (gait speed: *p* = 0.005, hand grip strength: *p* = 0.003, and leg strength: *p* = 0.0009; Table 1). FI-OutRef (*p* = 0.11) and levels of the chronic inflammation marker suPAR (*p* = 0.16) as well as levels of TNF-α (*p* = 0.76) and IL-18 (*p* = 0.48) were unchanged between admission and follow-up (Table 1).

Next, we compared characteristics of Patients at follow-up to Older Controls. Despite improvements since hospitalization, Patients at the follow-up visit were frailer (higher FI-OutRef, *p* < 0.0001), had higher CRP (*p* = 0.0003), IL-6 (*p* = 0.005), and GDF15 (*p* < 0.0001) levels and overall decreased physical function (gait speed: *p* < 0.0001, hand grip strength: *p* = 0.009, leg strength: *p* = 0.02) compared to the Older Controls (Table 1). In addition, the inflammatory markers suPAR (*p* < 0.0001), TNF-α (*p* = 0.005), and IL-18 (*p* = 0.02) were elevated in the Patient Group compared to the Older Controls (Table 1). Patients also had lower cognitive function compared to the Older Controls (Trail making test A *p* = 0.001, Trail making test B *p* < 0.0001), although not when assessed using the MMSE (*p* = 0.31). Four Patients had died within 1 year after the 30-day follow-up visit (causes of death were: renal insufficiency [*n* = 1], respiratory insufficiency [*n* = 1], cancer [*n* = 1], unknown [*n* = 1]), while all Older Controls were alive 1 year after their inclusion (*p* = 0.12). At the 1-year follow-up, Patients remained frailer than Older Controls (Additional file 2. Table S4).

We then assessed differences in baseline characteristics between Older and Young Controls. Young Controls had lower BMI (*p* = 0.0008) and lower levels of the inflammatory markers suPAR (*p* < 0.0001), CRP (*p* = 0.0002), IL-6 (*p* < 0.0001), IL-18 (*p* = 0.03) and TNF-α (*p* < 0.0001), as well as GDF15 (*p* < 0.0001) compared to Older Controls (Table 1). Young Controls also had better physical and cognitive function (*p* < 0.0001 for all tests) compared to the Older Controls (Table 1). FI-OutRef tended to be lower in the Young Controls compared to Older Controls, although this was not statistically significant (*p* = 0.07; Table 1).

Plasma levels of IL-1β and IL-10, were only detected in 7.3 and 11.8% of samples respectively and are therefore not included in the analyses. MIP-1α and IL-8 were only measured in samples from 13 Patients at follow-up, 13 Older Controls, and 14 Young Controls but were undetectable in all samples.

### Monocyte phenotypes

We first compared total monocyte numbers in each of the groups. Total monocyte numbers in the Patient Group were temporarily elevated at hospital admission (mean: 0.74 × 10^9^/L, 95% CI: 0.65 to 0.83) compared to 30-day follow-up (0.64 × 10^9^/L, 95% CI: 0.58 to 0.70; *p* = 0.03). Monocyte numbers of Patients at follow-up were not significantly different from that of Older Controls (0.58 × 10^9^/L, 95% CI: 0.53 to 0.63; *p* = 0.11). Young Controls had fewer total monocytes (0.48 × 10^9^/L, 95% CI: 0.45 to 0.53, *p* = 0.002) than Older Controls.

**Fig. 2:**
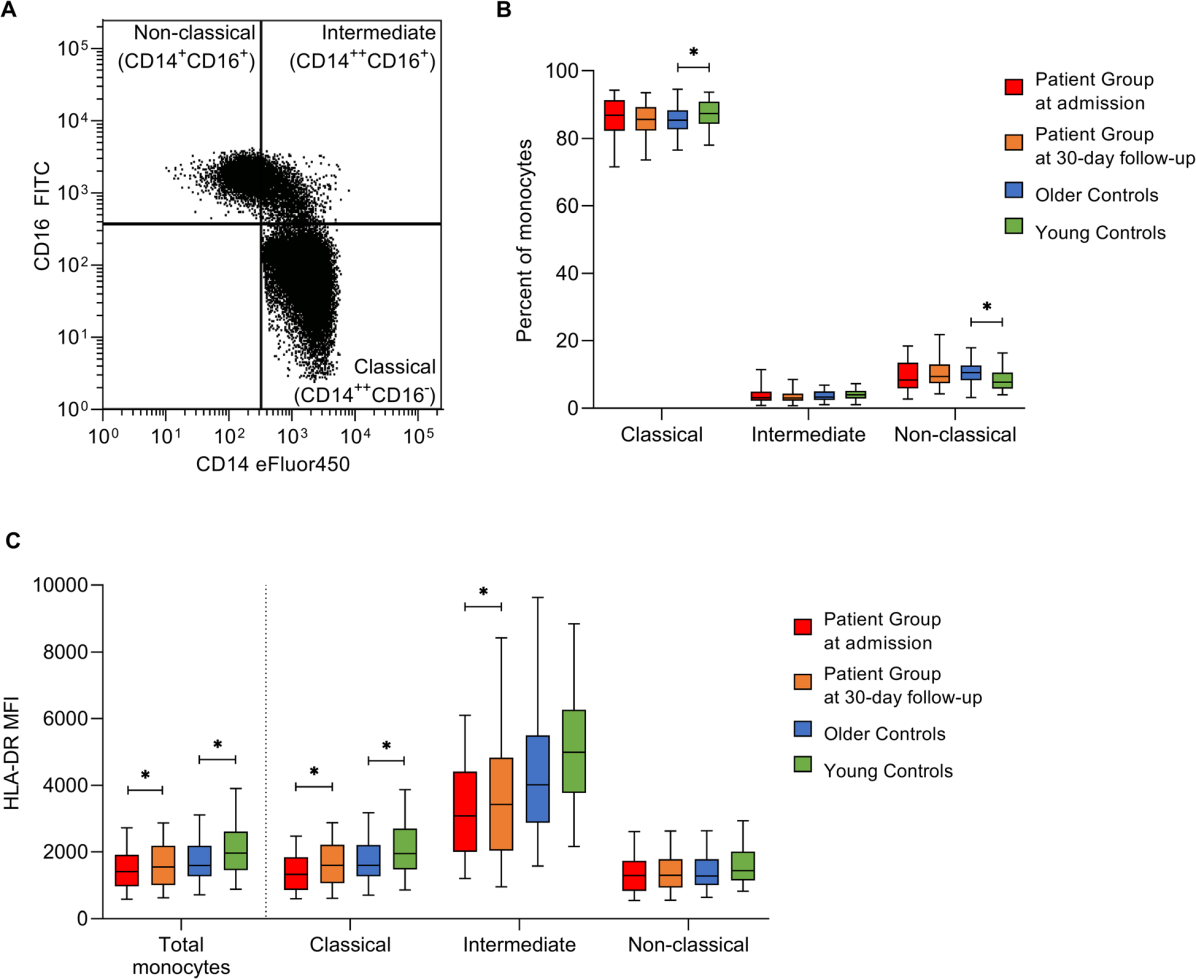
Monocyte subsets and HLA-DR expression. **a** Representative dot plot for the gating strategy for the CD14 CD16 monocyte subsets. **b** CD14 CD16 monocyte subset distribution in the Patient Group at admission (*n* = 42) and 30-day follow-up (*n* = 50), and Older (*n* = 52) and Young (*n* = 59) Control groups. Paired t-test was used for comparison between Patient Group and Older Controls, unpaired t-test was used for comparison between Older and Young Controls. **c** Total CD14 CD16 monocyte and subsets HLA-DR expression levels for the Patient Group at admission (*n* = 48 for total monocyte and 42 for subsets) and at the 30-day follow-up (*n* = 52 for total monocyte and 50 for subsets), and Older (*n* = 52) and Young (*n* = 59) Controls. Wilcoxon signed-rank test was used for comparisons between Patient Group at admission and 30-day follow-up, and between Patient Group at 30-day follow-up and Older Controls, Wilcoxon rank-sum test was used for comparison between Older and Young Controls. Box plots depict median and IQR, error bars indicate 95% CI. * indicates a *p*-value < 0.05

We then assessed monocyte subset distribution and HLA-DR expression in unstimulated whole blood samples using flow cytometry. An example of gating for monocyte subsets is presented in Fig. 2a.

In the Patient Group, monocyte subset distribution was unchanged between admission (mean percentage: classical: 86.0, 95% CI: 83.8 to 88.1; intermediate: 4.1, 95% CI: 3.2 to 5.1; non-classical: 9.3, 95% CI: 7.8 to 10.8) and 30-day follow-up (classical: 85.4, 95% CI: 83.7 to 87.2, *p* = 0.15; intermediate: 3.5, 95% CI: 2.6 to 4.1, *p* = 0.34; non-classical: 10.4, 95% CI: 9.0 to 11.8, *p* = 0.48; Fig. 2b). In the total monocyte population, HLA-DR expression was lower at admission (median HLA-DR MFI: 1244, IQR: 959 to 1897) compared to follow-up (MFI: 1544, IQR: 1027 to 2176; *p* = 0.02; Fig. 2c), which could be attributed to increased HLA-DR expression in the classical (*p* = 0.004) and intermediate subsets (*p* = 0.03).

We then compared Older Controls to the Patient Group at follow-up. There were no differences in subset distribution (classical: 85.5, 95% CI: 84.2 to 86.9, *p* = 0.63; intermediate: 3.7, 95% CI: 3.3 to 4.2, *p* = 0.77; non-classical: 10.4, 95% CI: 9.3 to 11.6, *p* = 0.70; Fig. 2b) or HLA-DR expression (MFI: 1593, IQR: 1273 to 2179, *p* = 0.42; Fig. 2c) of monocytes from Older Controls compared to the Patient Group.

Finally, we compared Older Controls to Young Controls. Young controls had fewer non-classical CD14^+^CD16^+^ monocytes (8.4, 95% CI: 7.5 to 9.3, *p* = 0.007) than Older Controls–and consequently a greater proportion of classical CD14^++^CD16^−^ monocytes (87.4, 95% CI: 86.2 to 88.6, *p* = 0.04; Fig. 2b) compared to the Older Controls. Monocytes from Young Controls also had higher HLA-DR expression (MFI: 1968, IQR: 1451 to 2612, *p* = 0.01; Fig. 2c) than monocytes from Older Controls, which could be attributed to increased HLA-DR expression in the classical subset (*p* = 0.01).

### Monocyte NF-κB signaling

**Fig. 3:**
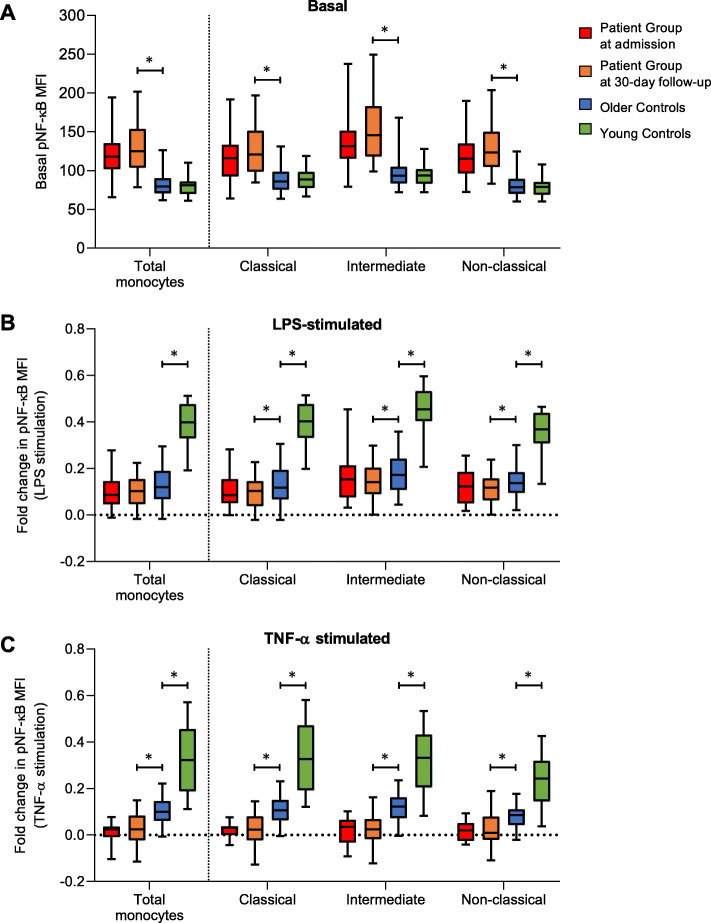
Basal and induced pNF-κB levels in monocytes. **a** Basal pNF-κB levels in monocytes from the Patient Group at admission (*n* = 46) and at follow-up (*n* = 52), Older Controls (*n* = 52) and Young Controls (*n* = 59). **b** Fold change in pNF-κB levels in monocytes from the Patient Group at admission (*n* = 36) and at follow-up (*n* = 50), Older Controls (*n* = 52) and Young Controls (*n* = 19) after stimulation with LPS for 15 min. **c** Fold change in pNF-κB levels in monocytes from the Patient Group at admission (*n* = 16) and at follow-up (*n* = 31), Older Controls (*n* = 52) and Young Controls (*n* = 19) after stimulation with TNF-α for 15 min. Wilcoxon signed-rank test was used for comparison between Patient Group at admission and 30-day follow-up and between Patient Group at 30-day follow-up and Older Controls, Wilcoxon rank-sum test was used for comparison between Older and Young Control groups. Box plots depict median and IQR, error bars indicate 95% CI. * indicates a *p*-value < 0.05

Next, we measured basal levels of NF-κB phosphorylation (pNF-κB; p65/RelA S529) and induction of NF-κB phosphorylation upon stimulation with LPS or TNF-α in total monocytes and their subsets (Fig. 3).

Within the Patient Group, basal pNF-κB levels at admission (pNF-κB MFI: 118, IQR: 102 to 135) were not significantly different from follow-up levels (MFI: 125, IQR: 105 to 153, *p* = 0.14; Fig. 3a). At admission, monocytes from Patients were able to significantly induce NF-κB phosphorylation in response to LPS (fold change: 0.10, 95% CI: 0.07 to 0.13, *p* < 0.0001, Fig. 3b) but not TNF-α (0.01, 95% CI: − 0.01 to 0.04, *p* = 0.20; Fig. 3c). There were no associations of basal pNF-κB levels or responses to LPS or TNF-α stimulations at admission with length of hospital stay or patient-reported reason for admission (data not shown). At follow-up, these responses (LPS: 0.10, 95% CI: 0.08 to 0.12, *p* < 0.0001; and TNF- α: 0.02, 95% CI: − 0.00 to 0.05, *p* = 0.07) were not significantly different compared to admission (*p* = 0.40; TNF-α: *p* = 0.42).

Compared to the Patient Group at follow-up, Older Controls had significantly lower basal pNF-κB levels (MFI: 80, IQR: 71 to 90, *p* < 0.0001; Fig. 3a), and the same trend was also observed within each of the monocyte subsets. Monocytes from Older Controls were able to significantly induce NF-κB phosphorylation in response to LPS (0.13, 95% CI: 0.10 to 0.15, *p* < 0.0001; Fig. 3b) and TNF-α stimulation (0.10, 95% CI: 0.08 to 0.12, *p* < 0.0001; Fig. 3c), and these responses were significantly greater than those from the Patient Group at follow-up (LPS: *p* = 0.05; TNF-α: *p* < 0.0001).

Compared to Older Controls, basal pNF-κB levels in monocytes were not different in Young Controls (MFI: 81, IQR: 70 to 86; *p* = 0.72; Fig. 3a). Monocytes from Young Controls were also able to significantly induce NF-κB phosphorylation in response to stimulation by LPS (0.40, 95% CI: 0.35 to 0.44, *p* < 0.0001; Fig. 3b) and TNF-α (0.33, 95% CI: 0.27 to 0.40, *p* < 0.0001; Fig. 3c), and these responses were significantly greater than those from Older Controls (LPS: *p* < 0.0001; TNF-α: *p* < 0.0001).

We also measured NLRP3 inflammasome expression in monocytes. NLRP3 expression followed the same pattern as basal NF-κB phosphorylation. NLRP3 expression was unchanged between admission (MFI: 57, IQR: 49 to 71) and follow-up in the Patient Group (MFI: 59, IQR: 47 to 71, *p* = 0.69, Additional File 3; Figure S1). Older Controls had lower NLRP3 levels (MFI: 45, IQR: 35 to 54, *p* < 0.0001) than the Patient Group at follow-up. Young Controls (MFI: 47, IQR: 38 to 55 *p* = 0.43; Additional File 3; Figure S1) had similar levels than Older Controls.

In all three groups, basal pNF-κB and NLRP3 levels were highest in the CD14^++^CD16^+^HLA-DR^high^ intermediate monocyte subset (Fig. 3a and Additional File 3; Figure S1). Induction of pNF-κB in response to LPS stimulation was significantly different within each monocyte subsets in all groups. The magnitude (high to low) of pNF-κB induction in response to LPS in each subset was as follows for the Patient Group at follow-up and Older Controls: intermediate > non-classical > classical, and for Young Controls: intermediate > classical > non-classical (Fig. 3b). The induction of pNF-κB in response to TNF-α stimulation was also significantly different within each monocyte subsets in Older and Young Controls. The magnitude of pNF-κB induction in response to TNF-α in each subset was as follows for Older Controls: intermediate > classical > non-classical, and for Young Controls: classical > intermediate > non-classical. No monocyte subset from the Patient Group could induce pNF-κB signaling in response to TNF-α stimulation (Fig. 3c).

### Relationships between monocyte basal pNF-κB, chronic inflammation, and aging measures

While Patients and Older Controls differed in the ability of their monocytes to induce pNF-κB in response to stimulation, they differed to a greater extend in their monocytes’ basal pNF-κB levels. Thus, we investigated associations of monocyte basal pNF-κB with chronic inflammation and aging measures in the Patient Group at the 30-day follow-up and in Older Controls.

First, we assessed whether age and sex influenced monocyte basal pNF-κB MFI in each group. In the Patient Group, monocyte basal pNF-κB MFI was not dependent on age (β: -0.41, 95% CI: − 1.5 to 0.7, *p* = 0.45) or sex (β: 1.76, 95% CI: − 12.5 to 18.4, *p* = 0.82). In Older Controls, monocyte pNF-κB MFI increased by 1.22% per year (95% CI: 0.5 to 2.0, *p* = 0.002) and was lower in men (β: -11.13, 95% CI: − 20.2 to − 1.0, *p* = 0.03). However, male participants were on average younger than female participants, and the difference between male and female participants became non-significant when adjusted for age (β: -8.48, 95% CI: − 17.5 to 1.5, *p* = 0.09).

In the Patient Group, there were no significant associations between monocyte pNF-κB MFI and any of the inflammation or aging measures. In the Older Controls, a 10% increase in monocyte basal pNF-κB MFI was significantly associated with a higher FI-OutRef and higher levels of the inflammatory markers IL-6 and TNF-α, and tended to be associated with higher levels of suPAR and decreased hand grip strength (Table 2). When adjusted for age, the association of pNF-κB MFI with FI-OutRef remained statistically significant (β: 0.23, 95% CI: 0.0 to 0.4, *p* = 0.02), while and the association of pNF-κB with TNF-α (β: 3.22, 95% CI: − 0.4 to 7.0, *p* = 0.08) became weaker and the association of pNF-κB with IL-6 was no longer statistically significant (β: 7.39, 95% CI: − 3.3 to 19.3, *p* = 0.18).

**Table 2:** Linear regressions for the associations of monocyte basal pNF-κB MFI with plasma biomarkers and aging measures in the Patient Group at 30-day follow-up and Older Controls

	Patient Group at 30-day follow-up			Older Controls		
	Estimate (β)	95% CI	*P*-value	Estimate (β)	95% CI	*P*-value
Plasma biomarkers						
suPAR (ng/mL) ^a^	1.48	(− 3.0–6.1)	0.51	3.16	(− 0.5–7.0)	0.09
CRP (mg/L) ^a^	5.96	(−7.3–21.2)	0.39	7.62	(− 6.0–23.3)	0.28
IL-6 (pg/mL) ^a^	3.92	(−4.4–13.0)	0.36	10.64	(0.4–21.9)	0.04
IL-18 (pg/mL) ^a^	3.46	(−2.9–10.2)	0.29	3.08	(− 3.5–10.1)	0.36
TNF-α (pg/mL) ^a^	2.72	(−3.3–9.1)	0.37	4.85	(1.4–8.5)	0.007
GDF15 (pg/mL) ^a^	2.97	(−2.9–9.1)	0.32	1.67	(− 2.8–6.3)	0.46
Frailty						
FI-OutRef	−0.05	(− 0.3–0.2)	0.71	0.24	(0.1–0.4)	0.005
Physical function						
4-m gait speed (m/s)	0.01	(−0.0–0.0)	0.35	0.00	(0.0–0.0)	0.80
Hand grip strength (kg)	− 0.02	(− 1.3–1.3)	0.97	−1.17	(− 2.5–0.2)	0.08
Chair stand test (repetitions)	0.29	(−0.2–0.8)	0.23	−0.30	(− 0.8–0.2)	0.23
Cognitive function						
MMSE (points)	−0.03	(− 0.3–0.2)	0.82	−0.19	(− 0.4–0.1)	0.16
Trail making test A (seconds)	−1.44	(− 6.2–3.5)	0.55	2.79	(−3.3–9.2)	0.37
Trail making test B (seconds)	−1.24	(− 6.9–4.8)	0.67	3.82	(− 3.0–11.1)	0.27

^a^ Variables were log-transformed, and estimates represent percent change in dependent variable per 10% increment in pNF-κB MFI

Abbreviations: *CRP* C-reactive protein, *FI-OutRef* Frailty index OutRef, *GDF15* growth differentiation factor 15, *IL* interleukin, *MMSE* Mini Mental State Examination, *suPAR* soluble urokinase plasminogen activator receptor, *TNF-α* tumor necrosis factor alpha

Because CMV has been shown to activate NF-κB in monocytes [32, 33], we also tested whether CMV IgG seropositivity was associated with pNF-κB levels. CMV IgG seropositivity was not associated with increased pNF-κB MFI in the Patient Group (β: 8.02, 95% CI: − 7.0 to 25.4, *p* = 0.31) nor for Older Controls (β: -3.52, 95% CI: − 14.8 to 9.3, *p* = 0.57).

Finally, we assessed the influence of monocyte NF-κB phosphorylation on the probability of belonging to the Patient Group (i.e. having a recent unplanned ED visit), as well as possible mediating or confounding effects of inflammation, frailty, physical and cognitive function, and CMV infection on this relationship. The odds of belonging to the Patient Group increased by 80% for each 10% increase of pNF-κB MFI (Table 3). We then adjusted the logistic regression model for each marker of inflammation, frailty, physical and cognitive function, and CMV infection, individually. The OR estimate was not affected by the adjustments, suggesting that none of the markers were mediators or confounders for the relationship between monocyte basal pNF-κB and belonging to the Patient Group. Of note, adjusting for GDF15 levels had the largest effect, by decreasing the odds of belonging to the Patient Group from 80% to 69% per 10% increase in pNF-κB MFI.

**Table 3:** Logistic regression for the probability of belonging to the Patient Group based on monocyte basal pNF-κB levels

	OR Estimate	95% CI	*P*-value
**Unadjusted logistic regression**	1.80	(1.3–2.4)	< 0.0001
**Adjusted for**			
Plasma biomarkers			
suPAR (ng/mL)	1.78	(1.3–2.5)	0.0004
CRP (mg/L)	1.75	(1.3–2.4)	0.0003
IL-6 (pg/mL)	1.77	(1.3–2.4)	0.0001
IL-18 (pg/mL)	1.86	(1.4–2.5)	0.0001
TNF-α (pg/mL)	1.82	(1.3–2.5)	0.0002
GDF15 (pg/mL)	1.69	(1.2–2.3)	0.0009
Frailty			
FI-OutRef	1.79	(1.2–2.6)	0.003
Physical function			
4-m gait speed (m/s)	1.81	(1.3–2.5)	0.0001
Hand grip strength (kg)	1.77	(1.3–2.4)	0.0002
Chair stand test (repetitions)	1.74	(1.3–2.4)	0.0005
Cognitive function			
MMSE (points)	1.76	(1.3–2.4)	0.0002
Trail making test A (seconds)	1.77	(1.3–2.4)	0.0004
Trail making test B (seconds)	1.99	(1.1–3.6)	0.02
CMV IgG seropositivity	1.82	(1.3–2.5)	< 0.0001

Abbreviations: *CMV* cytomegalovirus, *CRP* C-reactive protein, *FI-OutRef* Frailty index OutRef, *GDF15* growth differentiation factor 15, *IL* interleukin, *MMSE* Mini Mental State Examination, *suPAR* soluble urokinase plasminogen activator receptor, *TNF-α* tumor necrosis factor alpha

## Discussion

We assessed the effects of chronological age on monocyte NF-κB signaling as well as differences between older adults on different aging trajectories by comparing a group of recently hospitalized older Patients to age-matched Older Controls. Additionally, we assessed the relationships between monocyte NF-κB signaling and inflammation, frailty, and physical and cognitive function in older adults. Our results from comparisons between Young and Older Controls showed that a higher chronological age is characterized by alterations in monocyte phenotype and a decreased ability of monocytes to induce NF-κB signaling in response to LPS and TNF stimulation. The ability of monocytes to induce NF-κB signaling in response to stimulation was further reduced in the Patient Group compared to Older Controls, and in particular, monocytes from the Patient Group showed significant defects in their response to TNF-α induction of pNF-κB signaling. The Patient Group also had significantly elevated levels of inflammatory markers and elevated basal NF-κB phosphorylation levels compared to age-matched Older Controls (inflammaging). However, associations of basal NF-κB phosphorylation with inflammatory markers and frailty were weak.

We designed this study with the aim of including two groups of older individuals with different aging trajectories. Frailty and comorbidity are important risk factors for ED visits in older adults [34, 35]. Thus, we hypothesized that older adults requiring a visit to the ED would be a heterogeneous, frail population with comorbidities, and thereby show signs of accelerated aging compared to age-matched older adults without recent hospital admissions. Although participants in the Patient Group appeared to have recovered at the 30-day follow-up visit, they still had lower physical function, were frailer, and had elevated levels of inflammatory and senescence markers (CRP, IL-6, IL18, TNF-α, suPAR, and GDF15) [36] compared to age-matched Older Controls. Additionally, at the one-year follow-up, the Patients remained frailer than Older Controls and had elevated levels of inflammatory biomarkers and reduced physical and cognitive function. Furthermore, four Patients had died after 1 year, while all Older Controls were still alive. Therefore, we argue that the Patient Group exhibits accelerated aging compared to the age-matched Older Controls.

Several studies have shown that monocytes from older adults have a higher basal production of cytokines, including IL-1β, IL-6, IL-8, and TNF-α, compared to monocytes from young adults [19, 22, 23, 37, 38]. These cytokines are under transcriptional regulation of NF-κB, therefore, we, and others, hypothesized that increased NF-κB activity with age may be involved in the increased production of cytokines (and thereby contribute to inflammaging) [18]. We did not observe significant differences in basal levels of NF-κB phosphorylation between Older and Young Controls despite the clear differences in levels of inflammatory markers. Our results are in contrast with a study by Qian et al. who found higher levels of NF-κB in the nucleus of monocytes from older compared to young individuals [20]. This discrepancy could be due to differences in the method of quantification of NF-κB (i.e. nuclear localization versus p65 S529 phosphorylation). Non-classical CD14^+^CD16^+^ monocytes have a pro-inflammatory profile and accumulate with age, and were therefore suggested to contribute to chronic inflammation [19, 21, 23, 37, 39–41]. We observed accumulation of non-classical CD14^+^CD16^+^ monocytes in older compared to young adults, but basal pNF-κB levels did not differ in non-classical monocytes from older and young adults. Moreover, we found that intermediate monocytes had higher basal pNF-κB levels than non-classical monocytes. While there is evidence that unstimulated non-classical monocytes produce higher levels of certain cytokines than intermediate and classical monocytes, it is unclear whether it is a direct result of increased basal pNF-κB activity, or whether it is the result of increased activity of other pathways that are specifically upregulated in non-classical monocytes such as the MAPK/ERK pathway [39]. Furthermore, we only found weak associations of basal pNF-κB levels with IL-6 and TNF-α in older adults, challenging the role of S529 p65 NF-κB subunit phosphorylation in transactivation and upregulation of downstream inflammatory genes in older individuals. Many other pathways such as the JAK/STAT and MAPK/ERK play central roles in inflammatory signaling and may be involved in inflammaging [39, 42, 43]. In particular, activation (phosphorylation) of the JAK/STAT pathway in monocytes was more strongly associated with age and circulating cytokine levels than the NF-κB pathway in a recent study [42].

Monocytes from frail older adults have also been shown to produce elevated levels of cytokines compared to monocytes from non-frail older adults [44, 45]. In line with these observations, we found elevated monocyte basal pNF-κB and NLRP3 levels in older Patients with signs of accelerated aging compared to age-matched controls. It is unclear whether this is due to accumulation of inflammatory non-classical monocytes in frail older adults, we found both reports of unchanged and increased proportions of CD14^+^CD16^+^ monocytes in frail compared to non-frail older adults [46, 47]. Our results suggest that it is not the case, as we did not observe differences in monocyte subset distribution between Patients and age-matched Older Controls, furthermore, NF-κB and NLRP3 levels were elevated in all monocyte subsets. Inflammaging was recently proposed to result from the accumulation of damage-associated molecular patterns (DAMPs) [48, 49]. Patients had elevated FI-OutRef compared to Older controls, reflecting accumulation of organ damage which may cause elevated levels of DAMPs and result in elevated monocyte basal pNF-κB and NLRP3 levels. Furthermore, in the Older Controls, basal pNF-κB was associated with FI-OutRef.

Immune function declines with age, however, it is unclear how monocyte function is affected. Reports are conflicting, but a majority of studies has shown that monocytes of older adults produce reduced amounts of cytokines than those of young adults upon Toll-like receptor (TLR) stimulation [10, 19, 22, 23, 37, 38, 44, 50]. There are also reports of reduced activation of the MAPK/ERK and JAK/STAT pathways in response to TLR1/2 and cytokine stimulation in older adults [37, 43]. Qian et al. observed decreased total and phosphorylated p65 levels and reduced NF-κB nuclear translocation in response to TLR5 stimulation in monocytes from older compared to young individuals, despite a greater decrease in IκBα (inhibitor of kappa B) levels in older individuals [20]. This is in line with our results showing reduced pNF-κB induction in response to TLR4 stimulation in monocytes from Older compared to Young Controls. We also show a reduced induction to tumor necrosis factor receptor (TNF-R) stimulation, which has not been reported before. The only other study–to our knowledge–to investigate differences in monocyte NF-κB signaling in frail and non-frail older adults reported no difference in NF-κB (S529), p38 MAPK, and ERK signaling in HLA-DR + CD11c + CD14+ monocytes stimulated with LPS or R848 [47]. However, the authors did not report whether there were differences in basal levels of NF-κB phosphorylation. We used a higher concentration of LPS than the study by Compté et al. [47] and we were able to show that pNF-κB induction in response to stimulation is slightly reduced in the Patient Group compared to Older Controls. Furthermore, pNF-κB induction in response to TNF-R stimulation in monocytes from Patients was entirely blunted. Together, our results demonstrate that monocyte responsiveness decreases with chronological age (immunosenescence) and is further reduced in individuals with accelerated aging compared to age-matched controls. Responses to both LPS and TNF-α were affected. To the best of our knowledge, changes in TNF-R expression on monocytes with age has not yet been studied, and reports of changes in TLR4 expression are conflicting [20, 50, 51]. In addition to the potential effect of age on TLR and TNF-R expression, impaired NF- κB activation could involve impaired activity of molecules in the signaling pathways downstream of TLR4 and TNF-R*.* Thus, adding to the previous report of dysregulation of the NF-κB pathway with age demonstrated by Qian et al. [20], we showed that one mechanism for this dysregulation could involve defective phosphorylation of the Ser529 residue of p65. The only known kinase–to date–to phosphorylate the S529 residue is Casein kinase II (CK2) [16, 52, 53]. CK2 activity downregulation is associated with cellular senescence [54], and therefore further studies are needed to investigate if CK2 activity is affected by aging.

Expression of HLA-DR molecules on the surface of monocytes is crucial for their function as antigen presenting cells, linking innate and adaptive immune responses. Some studies have reported decreases in monocyte HLA-DR expression with age [21, 55], while others reported no change [56, 57]. Here, we show that HLA-DR expression on monocytes decreased with chronological age. This may have functional relevance, and along with our observation of decreased NF-κB induction upon stimulation in older adults, would provide an additional mechanism for decreased immune responsiveness in older compared to young individuals. To the best of our knowledge, HLA-DR expression has not yet been investigated in relation to frailty or accelerated aging. In our study, HLA-DR expression was not further reduced in the Patient Group compared to Older Controls, but was temporarily decreased during acute illness. This is in line with reports of decreased HLA-DR expression on monocytes during systemic inflammatory response syndrome (SIRS) and sepsis, reflecting a state of immune suppression [58–60].

Our study has some limitations. First, we assessed NF-κB signaling only by p65/RelA S529 phosphorylation. While S529 phosphorylation is necessary for transactivation activity in a gene-specific manner, it is only one of many phosphorylation sites distributed among many protein subunits required for full transcriptional activity of NF-κB [15, 16, 52, 61]. Furthermore, nuclear localization, which was not assessed, is also necessary for transcriptional activity. Because different phosphorylation sites, such as S536 or S276 are phosphorylated by different kinases and have different impacts on the function of NF-κB, a more comprehensive evaluation of NF-κB activity including nuclear localization and other phosphorylation sites may better reveal different associations of NF-κB activity with chronic inflammation and aging [15, 16, 52]. Second, although the Patient Group appeared to have recovered from the acute hospitalization with lower CRP and IL-6 levels and regained physical strength at the 30-day follow-up visit. It is possible that the recent hospitalization and acute illness had a confounding effect on the aging and immune measures, and we observed sustained NF-κB signaling between hospital admission and 30 days after discharge. In older adults admitted for acute illness, although recovery begins during hospitalization and mostly occurs within the first month after discharge, many patients are still recovering for several months [62–64]. Although Patients remained frailer than Older Controls after 1 year, we did not assess monocyte function after the 30-day follow-up and cannot exclude that NF-κB signaling in Patients could be restored to levels similar to that of Older Controls at a later time. Because patients were acutely admitted, we could not collect data on aging and immune measures prior to the onset of illness. This is a challenge for the design of studies involving acutely hospitalized older adults. Third, we did not collect information on diagnoses and number of comorbidities in the Patient Group and Older Controls and were therefore unable to adjust for them in our analyses.

## Conclusions

Our results did not provide clear evidence for the role of monocyte NF-κB (p65/RelA pS529) signaling in age-related chronic inflammation. However, altered monocyte NF-κB signaling may play a role in accelerated aging and frailty. Older chronological age was associated with decreased monocyte NF-κB activation upon TLR4 and TNF-R stimulation as well as a decreased expression of HLA-DR molecules. Monocyte responses were further reduced in individuals with signs of accelerated aging. These alterations may contribute to impaired immunity to infection and have implications for the success of immunization in older adults and in populations with accelerated aging. Further investigation of the pathways involved in reduced monocyte function with age may help finding strategies to restore function to innate immune cells.

## Supplementary information

**Additional file 1: Table S1–3.** Lists of diagnoses and medication used for exclusion criteria for Older and Young Controls.

**Additional file 2: Table S4.** Characteristics of Patients and Older Controls at 1-year follow-up.

**Additional file 3: Figure S1.** Basal NLRP3 levels in CD14 CD16 monocytes and subsets.

## Data Availability

The datasets generated and/or analyzed during the current study are not publicly available due to regulations set out by the Danish Data Protection Agency regarding data anonymization but are available from the corresponding author on reasonable request.

## Abbreviations

BMI: Body mass index
CRP: C-reactive protein
CMV: Cytomegalovirus
DAMPs: Damage-associated molecular patterns
ED: Emergency department
ELISA: Enzyme linked immunosorbent assay
FI-OutRef: Frailty index OutRef
GDF15: Growth differentiation factor 15
IκBα: Inhibitor of kappa B
IL: Interleukin
LPS: Lipopolysaccharide
MIP-1α: Macrophage inflammatory protein-1α
MFI: Median fluorescence intensity
MMSE: Mini mental state examination
NF-κB: Nuclear factor kappa-light-chain-enhancer of activated B cells
NLRP3: NOD-, LRR- and pyrin domain-containing protein 3
OR: Odds ratio
pNF-κB: Phosphorylated NF-κB
SASP: Senescence-associated secretory phenotype (SASP)
SIRS: Systematic inflammatory response syndrome
suPAR: Soluble urokinase plasminogen activator receptor
TLR: Toll-like receptor
TNF-R: Tumor necrosis factor receptor
TNF-α: Tumor necrosis factor-α
