# Supplementary material for: Alterations of monocyte NF-κB p65/RelA signaling in a cohort of older medical patients, age-matched controls, and healthy young adults
Source: Immun Ageing. 2020 Sep 4;17:25. doi: 10.1186/s12979-020-00197-7 (PMC7938715; doi:10.1186/s12979-020-00197-7)
Supplement: Supplementary file 1 — Additional file 1: Table S1–3. Lists of diagnoses and medication used for exclusion criteria for Older and Young Controls. [file 12979_2020_197_MOESM1_ESM.docx]

**Additional file 1.** Lists of diagnoses and medications used for exclusion criteria for Older and Young Controls

**Table S1.** List of diagnoses used as exclusion criteria for Older Controls (ICD-10 codes)

| DC00 to 96 | Cancer diagnoses |
| --- | --- |
| DE10 | Type 1-diabetes |
| DE100 | Type 1-diabetes with coma |
| DE101 | Type 1-diabetes with ketoacidosis |
| DE102 | Type 1-diabetes with renal complications |
| DE103 | Type 1-diabetes with ophthalmic complications |
| DE104 | Type 1-diabetes with neurological complications |
| DE105 | Type 1-diabetes with peripheral circulatory complications |
| DE105A | Type 1-diabetes with peripheral angiopathy |
| DE105B | Type 1-diabetes with foot ulcer |
| DE105C | Type 1-diabetes with gangrene |
| DE105D | Type 1-diabetes with microangiopathy |
| DE106 | Type 1-diabetes with other specified complications |
| DE107 | Type 1-diabetes with multiple complications |
| DE108 | Type 1-diabetes with unspecified complications |
| DE109 | Type 1-diabetes without complications |
| DE109A | Type 1-diabetes UNS |
| DE271 | Primary adrenocortical insufficiency |
| DE271A | Addison’s disease |
| DE271B | Autoimmune adrenalitis |
| DG040 | Acute disseminated encephalitis |
| DG048 | Other encephalitis, myelitis and encephalomyelitis |
| DG048A | Postinfectious encephalitis NOS |
| DG048B | Postinfectious encephalomyelitis NOS |
| DG048C | Postinfectious myelitis NOS |
| DG049 | Encephalitis, myelitis and encephalomyelitis, unspecified |
| DG049A | Encephalitis UNS |
| DG049B | Encephalomyelitis UNS |
| DG049C | Myelitis UNS |
| DG049D | Cerebral ventriculitis UNS |
| DK51 | Ulcerative colitis |
| DK510 | Ulcerative pancolitis |
| DK512 | Ulcerative proctitis |
| DK513 | Ulcerative rectosigmoiditis |
| DK514 | Pseudopolyposis of colon |
| DK515 | Left sided colitis |
| DK515A | Left sided proctocolitis |
| DK515B | Left sided hemicolitis |
| DK518 | Other ulcerative colitis |
| DK518B | Other ulcerative colitis with intestinal obstruction |
| DK519 | Ulcerative colitis UNS |
| DK732 | Chronic active hepatitis NEC |
| DK732B | Type 1 autoimmune hepatitis with smooth muscle antibodies |
| DK732C | Type 2 autoimmune hepatitis without smooth muscle antibodies |
| DK732D | Autoimmune giant cell hepatitis |
| DK732E | Autoimmune hepatitis with primary biliary cirrhosis |
| DK732F | Autoimmune hepatitis with primary sclerosing cholangitis |
| DK732G | Autoimmune hepatitis UNS |
| DE063 | Autoimmune thyroiditis |
| DE063A | Hashimoto's thyroiditis |
| DE063B | Struma lymphomatosa |
| DK50 | Crohn disease |
| DK500 | Crohn disease of small intestine |
| DK500A | Crohn disease of duodenum |
| DK500B | Crohn disease of ileum |
| DK500C | Crohn disease of jejunum |
| DK500D | Terminal ileitis |
| DK501 | Crohn disease of large intestine |
| DK501D | Crohn disease of rectum |
| DK508 | Other Crohn disease |
| DK508A | Crohn disease of both small and large intestine |
| DK508C | Crohn disease with oral manifestation |
| DK508D | Crohn disease with ileocecal localisation |
| DK509 | Crohn disease UNS |
| DK900 | Coeliac disease |
| DK900B | Nontropical sprue |
| DL95 | Vasculitis limited to skin NEC |
| DL958 | Other vasculitis limited to skin |
| DL958A | Leukocytoclastic vasculitis |
| DL958B | Urticarial vasculitis |
| DM050 | Felty syndrome |
| DM31 | Other necrotizing vasculopathies |
| DM310 | Hypersensitivity angiitis |
| DM310A | Goodpasture syndrome |
| DM311 | Thrombotic microangiopathy |
| DM311A | Thrombotic thrombocytopenic purpura |
| DM311B | Buerger's disease |
| DM312 | Lethal midline granuloma |
| DM313 | Wegener's granulomatosis. |
| DM313A | Necrotizing respiratory granulomatosis |
| DM314 | Aortic arch syndrome |
| DM315 | Giant cell arteritis with polymyalgia rheumatica |
| DM315A | Temporal arteritis with polymyalgia rheumatica |
| DM316 | Other giant cell arteritis |
| DM316A | Temporal arteritis without polymyalgia rheumatica |
| DM317 | Microscopic polyangiitis |
| DM318 | Other specified necrotizing vasculopathies |
| DM318A | Hypocomplementaemic vasculitis |
| DM319 | Necrotizing vasculopathy UNS |
| DE050 | Thyrotoxicosis with diffuse goiter |
| DG610 | Guillain-Barré syndrome |
| DD58 | Other hereditary haemolytic anaemias |
| DD580 | Hereditary spherocytosis |
| DD581 | Hereditary elliptocytosis |
| DD581A | Congenital elliptocytosis |
| DD582 | Other haemoglobinopathies |
| DD582A | Abnormal haemoglobin NOS |
| DD582B | Congenital Heinz body anaemia |
| DD582C | Hb-C disease |
| DD582D | Hb-D disease |
| DD582E | Hb-E disease |
| DD588 | Other specified hereditary haemolytic anaemias |
| DD588A | Stomatocytosis |
| DD589 | Hereditary haemolytic anaemia UNS |
| DD590 | Drug-induced autoimmune haemolytic anaemia |
| DD591 | Other autoimmune haemolytic anaemias |
| DD591A | Chronic cold haemagglutinin disease |
| DG700 | Myasthenia gravis |
| DG702 | Congenital and developmental myasthenia |
| DG731 | Eaton-Lambert syndrome |
| DP940 | Transient neonatal myasthenia gravis |
| DG359 | Disseminated sclerosis UNS |
| DG359A | Relapsing-remitting disseminated sclerosis |
| DG359B | Primary disseminated sclerosis |
| DG359C | Progressive disseminated sclerosis |
| DD51 | Vitamin B12 deficiency anaemia |
| DD510 | Vitamin B12 deficiency anaemia due to intrinsic factor deficiency |
| DD511 | Vitamin B12 deficiency anaemia due to selective vitamin B12 malabsorption with proteinuria |
| DD512 | Transcobalamin II deficiency |
| DD513 | Other dietary vitamin B12 deficiency anaemia |
| DD518 | Other vitamin B12 deficiency anaemias |
| DD519 | Vitamin B12 deficiency anaemia UNS |
| DM941 | Relapsing polychondritis |
| DL40 | Psoriasis |
| DL400 | Psoriasis vulgaris |
| DL400B | Nummular psoriasis |
| DL400C | Plaque psoriasis |
| DL400D | Discoid psoriasis |
| DL401 | Generalized pustular psoriasis |
| DL401B | Impetigo herpetiformis |
| DL402 | Acrodermatitis continua |
| DL402B | Acrodermatitis continua maligna |
| DL403 | Pustulosis palmaris et plantaris |
| DL403A | Pustulosis palmaris |
| DL403B | Pustulosis plantaris |
| DL404 | Guttate psoriasis |
| DL405 | Arthropathic psoriasis |
| DL408 | Other psoriasis |
| DL408A | Psoriasis inversa |
| DL408B | Erythroderma psoriatica |
| DL408C | Psoriasis of the hand and feet |
| DL408D | Psoriasis of the nail |
| DL408E | Pityriasis amiantacea |
| DL409 | Psoriasis UNS |
| DM070 | Distal interphalangeal psoriatic arthropathy |
| DM071 | Arthritis mutilans |
| DM073 | Other forms of psoriatic arthropathies |
| DM073A | Psoriatic arthropathies UNS |
| DM073B | Other psoriatic arthropathies |
| DM05 | Seropositive rheumatoid arthritis |
| DM051 | Rheumatoid arthritis with lung disease |
| DM051A | Rheumatoid arthritis with Caplan syndrome |
| DM051B | Rheumatoid arthritis with pleural effusion |
| DM051C | Rheumatoid arthritis with interstitial diffuse lung fibrosis |
| DM051D | Rheumatoid arthritis with pleuritis |
| DM051E | Rheumatoid arthritis with rheumatoid nodules in the lung |
| DM051F | Rheumatoid arthritis with granulomas in the lung |
| DM053 | Rheumatoid arthritis with involvement of other organs and systems |
| DM058 | Other seropositive rheumatoid arthritis |
| DM059 | Seropositive rheumatoid arthritis UNS |
| DM06 | Other rheumatoid arthritis |
| DM060 | Seronegative rheumatoid arthritis |
| DM068 | Other specified rheumatoid arthritis |
| DM069 | Rheumatoid arthritis UNS |
| DM32 | Systemic lupus erythematosus |
| DM320 | Drug-induced systemic lupus erythematosus |
| DM321 | Systemic lupus erythematosus with organ or system involvement |
| DM328 | Other forms of systemic lupus erythematosus |
| DM329 | Systemic lupus erythematosus UNS |
| DM350 | Sjögren's syndrome |
| DM350A | Sjögren's syndrome with keratoconjunctivitis |
| DM350B | Sjögren's syndrome with lung involvement |
| DM350C | Sjögren's syndrome with xerostomia |
| DM350D | Sjögren's syndrome with myopathy |
| DM350E | Sjögren's syndrome with renal tubulo-interstitial disorders |

**Table S2.** List of diagnoses used as exclusion criteria for Young Controls

| Crohn disease |
| --- |
| Multiple sclerosis |
| Psoriasis |
| Rheumatoid arthritis |
| Systemic lupus erythematosus |
| Type I diabetes |
| Ulcerative colitis |
| Addison’s disease |
| Acute disseminated encephalitis |
| Autoimmune hepatitis |
| Autoimmune thyroiditis |
| Coeliac disease |
| Felty’s syndrome |
| Granulomatosis with polyangiitis |
| Thyrotoxicosis with diffuse goiter (Graves’ disease) |
| Guillain-Barré syndrome |
| Hashimoto's thyroiditis |
| Haemolytic anaemia |
| Eaton-Lambert syndrome |
| Myasthenia gravis |
| Pernicious anemia |
| Polychondritis |
| Sjögren's syndrome |
| Vasculitis |

**Table S3.** List of medications used as exclusion criteria for Older and Young Controls

| Prednisolone |
| --- |
| Cortisone (hydrocortisone, fludrocortisone) |
| Aspirin (acetylsalicylic acid, Kodimagnyl) |
| Ibuprofen |
| Infliximab (Remicade, Remsima, Inflectra) |
| Ciclosporine |
| Methotrexate |
