# Supplementary material for: Alterations of monocyte NF-κB p65/RelA signaling in a cohort of older medical patients, age-matched controls, and healthy young adults
Source: Immun Ageing. 2020 Sep 4;17:25. doi: 10.1186/s12979-020-00197-7 (PMC7938715; doi:10.1186/s12979-020-00197-7)
Supplement: Supplementary file 2 — Additional file 2: Table S4. Characteristics of Patients and Older Controls at 1-year follow-up. [file 12979_2020_197_MOESM2_ESM.docx]

**Additional file 2.** Characteristics of Patients and Older Controls at 1-year follow-up

**Table S4.** Characteristics of Patients and Older Controls at 1-year follow-up

|  | **Patient Group** | | **Older Controls** | |  |
| --- | --- | --- | --- | --- | --- |
|  | n | median (IQR) or n (%) | n | median (IQR) or n (%) | P value |
| **Demographics and lifestyle** |  |  |  |  |  |
| Age (years) ^1^ | 43 | 75.3 (70.8 – 81.1) | 48 | 75.9 (71.9 – 82.1) | n.s. |
| Sex (women) ^2^ | 43 | 22 (51.2) | 48 | 22 (45.8) | n.s. |
| BMI (kg/m^2^) ^1^ | 43 | 27.1 (23.5 – 30.7) | 47 | 25.9 (22.5 – 28.0) | n.s. |
| **Plasma biomarkers** |  |  |  |  |  |
| CRP (mg/L) ^3^ | 43 | 1.9 (0.9 – 4.9) | 47 | 1.3 (0.5 – 2.4) | 0.007 |
| suPAR (ng/mL) ^3^ | 43 | 3.0 (2.4 – 3.7) | 47 | 3.3 (2.8 – 4.1) | n.s. |
| IL-6 (pg/mL) ^3^ | 43 | 0.8 (0.5 – 1.4) | 47 | 0.6 (0.3 – 0.9) | 0.01 |
| IL-18 (pg/mL) ^3^ | 43 | 257.0 (210.5 – 376.1) | 47 | 224.1 (175.0 – 286.9) | n.s. |
| TNF-α (pg/mL) ^3^ | 43 | 9.1 (6.8 – 11.5) | 47 | 8.0 (6.6 – 10.3) | n.s. |
| GDF15 (pg/mL) ^3^ | 43 | 1377.1 (1051.4 – 1929.4) | 47 | 1090.0 (838.4 – 1506.3) | 0.02 |
| **Frailty** |  |  |  |  |  |
| FI-OutRef ^3^ | 43 | 1.1 (0.0 – 4.3) | 47 | 1.0 (0.0 – 2.0) | 0.01 |
| **Physical** **function** |  |  |  |  |  |
| 4-meters gait speed (m/s) ^1^ | 41 | 0.9 (0.7 – 1.0) | 48 | 1.2 (1.1 – 1.3) | <0.0001 |
| Hand grip strength (kg) ^1^ | 42 | 24.7 (18.6 – 35.2) | 48 | 28.8 (21.1 – 39.2) | n.s. |
| Chair stand test (repetitions) ^1^ | 32 | 13.0 (9.0 – 14.0) | 48 | 14.5 (12.5 – 18.0) | 0.006 |
| **Cognitive** **function** |  |  |  |  |  |
| MMSE (points) ^1^ | 41 | 29 (28 – 30) | 41 | 30 (28 – 30) | n.s. |
| Trail making test A (seconds) ^3^ | 39 | 40 (34 – 55) | 47 | 34 (30 – 50) | 0.03 |
| Trail making test B (seconds) ^3^ | 39 | 118 (86 – 225) | 46 | 97.5 (59 – 120) | 0.007 |

Abbreviations: BMI: body mass index; CRP: C-reactive protein; FI-OutRef: frailty index OutRef; GDF15: growth differentiation factor 15; IL: interleukin; MMSE: mini mental state examination; suPAR: soluble urokinase plasminogen activator receptor; TNF-α: tumor necrosis factor-alpha.

^1^ Student's unpaired t-test

^2^ Chi-square test

^3^ Wilcoxon rank-sum test
