# Supplementary material for: Alterations of monocyte NF-κB p65/RelA signaling in a cohort of older medical patients, age-matched controls, and healthy young adults
Source: Immun Ageing. 2020 Sep 4;17:25. doi: 10.1186/s12979-020-00197-7 (PMC7938715; doi:10.1186/s12979-020-00197-7)
Supplement: Supplementary file 3 — Additional file 3: Figure S1. Basal NLRP3 levels in CD14 CD16 monocytes and subsets. [file 12979_2020_197_MOESM3_ESM.docx]

**Additional file 3**

**
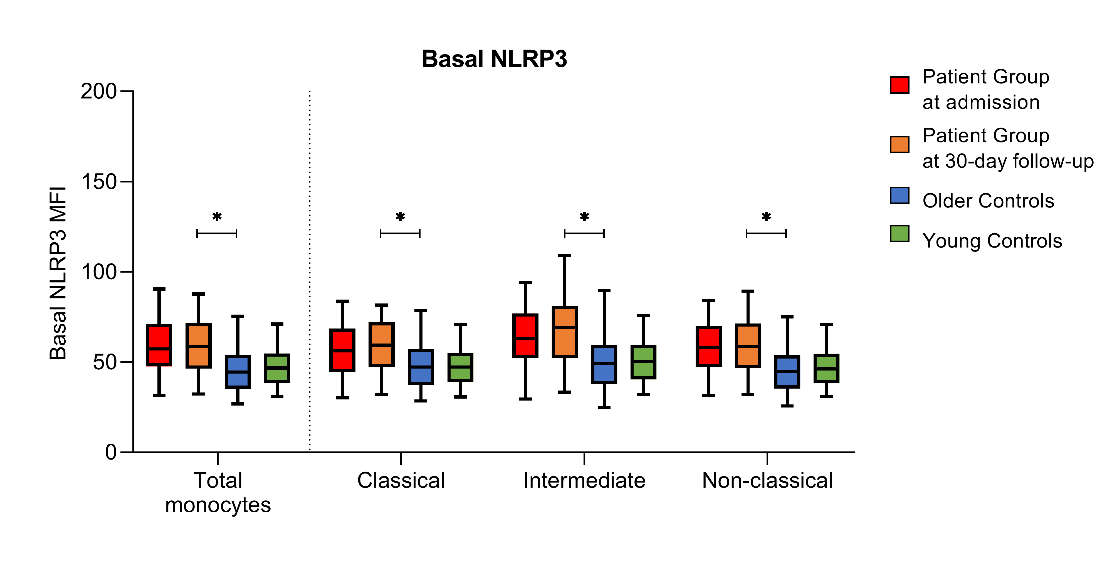
**

**Figure S1.** Basal NLRP3 levels in CD14 CD16 monocytes and their subsets.

Basal NLRP3 levels for the Patients at admission (n=48 for total monocytes and 42 for monocyte subsets) and at 30-day follow-up (n=52 for total monocytes and 50 for monocyte subsets), Older Controls (n=52) and Young Controls (n=59). Wilcoxon signed-rank test was used for comparison between Patients and Older Controls, Wilcoxon rank-sum test was used for comparison between Older and Young Control groups. Box plots depict median and IQR, error bars indicate 95% CI. * indicates a p-value <0.05.
